# Tuberculosis screening in household contacts of pulmonary tuberculosis patients in an urban setting

**DOI:** 10.1371/journal.pone.0240594

**Published:** 2020-10-15

**Authors:** Banurekha Velayutham, Lavanya Jayabal, Basilea Watson, Saraswathy Jagadeesan, Dhanalakshmi Angamuthu, Priscilla Rebecca, Bella Devaleenal, Dina Nair, Srikanth Tripathy, Sriram Selvaraju

**Affiliations:** 1 ICMR- National Institute for Research in Tuberculosis, Chennai, India; 2 National Tuberculosis Elimination Programme, Chennai, India; Jamia Hamdard, INDIA

## Abstract

**Background:**

Household contacts (HHC) of tuberculosis (TB) patients are at risk of TB infection and disease. The study assessed the utility of “Household contact card and register” for screening of HHC of pulmonary TB (PTB) patients for TB and explored the reasons for HHC not being screened and followed-up.

**Methods:**

The “Household contact card and register” was implemented by the Health Care Workers (HCW) of the TB Control Programme in Chennai District for screening HHC of index PTB patients initiated on treatment between June and August, 2018. Contacts were required to be screened within 2 months of treatment initiation of the index patient. Details collected included age, gender, smoking, alcohol use, immunosuppressive conditions and TB treatment. Symptom screening along with chest radiograph and or sputum examination was attempted. Follow-up TB screening at 6 and 12 months were performed. Screening of HHC was compared pre and post implementation phase. Proportions were computed for the data analysed.

**Results:**

HHC information was documented for 93% (1268/1364) of Index PTB patients. The main reasons of non-listing of HHC in 96 PTB patients were HCW non-availability or non-co-operation of the HHC. There were 2150 (80%) contacts who were screened for TB. Inconvenient time, feeling healthy, stigma, out-station visit were the main reasons for 537 contacts not undergoing TB screening. Anti-TB treatment was initiated in 21 (1%) of contacts diagnosed with TB. Preventive therapy was initiated in 59% (81/138) of contacts aged <6 years. The screening of HHC improved from 36% to 80% during the implementation phase. Follow-up TB screening at 12 months was performed in 50% of HHC and 2 incident TB cases were identified.

**Conclusion:**

“Household contact card and register” is a useful tool for HCWs for TB screening in HHC of PTB patients. Reasons for non-adherence to contact screening needs to be addressed.

## Introduction

The patient-centered care component of End tuberculosis (TB) strategy recommends systematic TB screening in high risk groups [1]. Household contacts (HHC) of TB patients are at a higher risk of TB infection and disease due to prolonged and proximal exposure to source TB case [2]. The National Tuberculosis Elimination programme (formerly known as Revised National Tuberculosis Control programme) recommends TB screening for all HHC of TB patients [3]. Screening of HHC of TB patients under TB Program settings has been sub-optimal [4–6].

Our previous study under TB program settings had reported that with training of Health care workers (HCW) and introduction of specific documentation (Isoniazid Preventive Therapy (IPT) card and register), implementation of IPT for child contacts improved from 19% to 61%. [7]. Currently, the treatment card of TB patients has provision to document the aggregate number of HHC identified, screened, evaluated for TB, diagnosed with TB and started on TB treatment categorized as aged <6 and >6 years to ensure HHC screening [3]. However, there is no specific systematic documentation of the process of detailed TB screening with line-listing of HHC which ensures better accountability. Moreover, details of risk factors for TB among HHC that requires special attention and close monitoring is not routinely captured. It has been reported earlier that majority of cases of active TB among HHC occurred within one year of source case’s diagnosis [2]. Hence, there is a need to follow-up the HHC to understand the occurrence of TB among them. However, the feasibility of follow-up of HHC under program settings has to be explored. Considering the importance of contact screening, in an attempt to prioritize this task under routine TB program settings, we attempted to assess the utility and feasibility of “Household contact card and register” for screening and follow-up of HHC of pulmonary TB (PTB) patients. We explored the reasons for HHC not being screened and followed-up. In addition, we describe the profile of HHCs and the investigations performed by HCWs to rule out TB in HHCs.

## Material and methods

This prospective cohort study was conducted in all the Tuberculosis Units (TU) (36 nos.) in Chennai District which is an urban setting. TU is the sub-district supervisory Unit under the TB Program covering a population of 2.5 lakhs. The study duration was from April, 2018 to December, 2019. Retrospective record review of HHC was done for PTB patients initiated on treatment from January to March, 2018 for pre-implementation data. During the implementation phase, HHC of Index PTB patients initiated on treatment from June to August, 2018 were considered for baseline screening. Subsequently, the follow-up period for HHC was 12 months upto October 2019.

### Compilation of baseline data on TB contact screening prior to usage of household contact card

The TB treatment cards of PTB patients initiated on treatment for a period of 3 months (January to March, 2018) were perused by the study staff prior to training of TB Program staff and implementation of HHC card. Index case details recorded include age, gender, type of TB case and sputum smear status. The HHC details documented include number of HHC, number screened, number with symptoms, number evaluated, number diagnosed, number put on treatment and number on Isoniazid preventive therapy (aged <6 years).

### Training of TB Program staff

The Medical Officer, TB—Health Visitor (TB-HV), Senior Tuberculosis Supervisor (STS) of the TB program were trained on the information to be captured in the Household contact card and register. The HCWs were informed to follow the routine TB screening procedures under the TB program for the household contacts [3].

### Description of Household contact card and register

‘Household contact card and register’ was designed to collect the following information by TB-HV / DOT provider

- Index patient details: Name, TB No, MDR treatment, date of start of treatment, sputum smear status, total number of HHC
- HHC details: Contact screening number, name, age, gender, mobile/landline number, body weight and height, known co-morbidity—HIV, diabetes, immunosuppressive therapy, current pregnancy status in female, habits—current smoking, alcohol use, current and past TB treatment

TB screening details to be documented by Medical Officer (At baseline and during follow-up) include: date of screening, presence of TB symptoms, details of chest x-ray, microbiology and other investigations, outcome of TB screening, details of TB treatment initiation and IPT initiation (at baseline TB screening only).

The TB-HV and STS maintained the HHC card and register respectively. The HHC card and register has been included as supporting information (S1 and S2 Files).

### Implementation of Household contact card and register

The TB-HV/DOT provider attempted to sensitize the TB patient on contact screening in the DOT centre within 2 weeks of treatment initiation. The importance of contact screening was explained and patient was instructed to bring the HHC for TB screening.

The TB-HV/DOT provider gave a unique contact screening number and collected the relevant information as mentioned earlier from the HHC (aged ≥18years) and from the parent or guardian (for HHC aged <18years). The height and weight of the HHC was recorded. Subsequently, the screening of HHC as per the TB program guidelines was routinely followed by the HCWs. The screening procedures were as follows: The HHC were enquired about symptoms suggestive of presumptive pulmonary and Extra-pulmonary TB. They were advised to take chest x-ray if not taken in the past one month. In addition, they were requested to provide 2 sputum samples for examination by smear/ CBNAAT (Xpert MTB/RIF). Children were referred to pediatric OPD in tertiary care hospitals to rule out TB as per the routine procedure.

If the HHC were diagnosed with TB, they were offered treatment as per TB program guidelines. TB-HV/DOT provider ensured that the relevant details were entered in the HHC card by the Medical Officer.

The follow-up of HHC not diagnosed with TB was at 6 and 12 months after baseline screening. TB symptom screening was done and further investigations as per the TB diagnostic algorithm were performed in case of symptoms.

Attempts were made to screen the HHC within two months of TB treatment initiation in the Index patient and the scheduled 6^th^ and 12^th^ month follow-up. Reminders through phone calls / home visits were done. The STS supervised the contact screening activities and documented the same in the HHC register.

### Focus group discussions (FGDs)

Three FGDs, were conducted by a facilitator, among STS and TB-HVs involved in HHC screening and supervision. There were about 10 participants in each FGD. The FGD guide was pre-tested prior to its use. This focussed on the

- Usefulness of HHC registration with a separate card/ register for TB screening and follow-up of HHC
- Reasons why HHC do not come for TB screening
- Reasons why HHC do not attend for follow-up
- Suggestions to improve HHC card and register for ease of use
- Suggestions to improve screening for TB in HHC

The responses were audio recorded and later transcribed.

### Operational definition

#### Index pulmonary TB (PTB) patient

A person of any age from a household who is the first case of new or recurrent pulmonary TB initiated on treatment under TB program in Chennai District during the study period.

#### Household contact

A person living with and sharing food from the same kitchen as the index patient for a minimum of three months prior to diagnosis of TB disease of the index case [8].

#### Household contact screened

A HHC was considered to have undergone TB screening if TB symptom screening has been done at the least.

#### Household contact diagnosed with TB

A HHC who is diagnosed with active TB (microbiologically confirmed active TB or as clinically diagnosed TB) as per TB program guidelines [3].

### Statistical considerations

Data from the HHC card was entered in the RedCap software. About 10% of the data were verified by the study team. Data was analysed using STAT Ver. 16.0.–Stata Corp., June 2016, USA.

Comparison of the proportion of HHC identified, screened, evaluated for TB, diagnosed with TB, initiated on TB treatment and HHC aged < 6 years initiated on IPT prior to and after implementation of HHC card was done using χ^2^ test. p- value <0.05 was considered statistically significant. Reasons for HHC not being screened for TB were quantified. Issues among key discussion topics were identified in FGD analysis.

### Ethical considerations

The study was approved by the National Institute for Research in Tuberculosis (NIRT)-Institutional Ethics Committee. Written informed consent was obtained from study participants prior to FGDs.

## Results

### Household contact identification and screening during the pre and post-implementation phase

There was an increase in the identification and documentation of HHC from 83% in the pre-implementation to 93% during implementation phase (Table 1). The screening of HHC improved from 36% to 80%. Those diagnosed with TB among those screened was 1% during implementation phase as compared to 4% in the pre-implementation phase. Treatment initiation was 100% in those diagnosed with TB during implementation phase as compared to 35% during pre-implementation. There was an increase in the proportion of HHC aged <6 years initiated on IPT from 34% to 59% during the implementation phase.

**Table 1:** Comparison of screening for tuberculosis in household contacts of pulmonary TB (PTB) patients pre and post ‘Household contact card’ implementation.

Household contact screening	Pre-implementation phase	Post-Implementation phase	p-value^
	(January to March, 2018)	(June to August, 2018)	
Index PTB patients started on treatment (n)	1236	1364	
Index PTB patients with documentation of household contacts	1023 (83%)	1268 (93%)	<0.001
	(95%CI: 81, 85)	(95%CI: 92, 94)	
Household contacts identified (n)	2773	2687	
Contacts screened	1001 (36%)	2150 (80%)	<0.001
	(95%CI: 34, 38)	(95%CI: 78, 81)	
Contacts diagnosed with TB	40 (4%)	21 (1%)	<0.001
	(95%CI: 3, 5)	(95%CI: 0.6, 1.5)	
Contacts started anti-TB treatment	14 (35%)	21 (100%)	<0.001
	(95%CI: 21, 52)	(95%CI: 84, 100)	
Contacts aged <6 years of smear-positive Index PTB patients (n)	202	138	
Contacts <6 years started on Isoniazid preventive therapy	69 (34%)	81 (59%)	<0.001
	(95%CI: 28, 41)	(95%CI: 50, 67)	

^Chi-square test.

95% CI: 95% Confidence Interval.

### Index case details

Of the 1268 index PTB patients with listed HHC, the median (IQR) contacts was 2 (1,3). There were 1103 (87%) index PTB patients who were sputum smear positive and 25 (2%) were on MDR-TB treatment.

### Characteristics of household contacts screened for TB

Of the 2693 HHC identified during the intervention phase, there were 40% males, median age was 30 years and median BMI was 22 Kg/m^2^ (Table 2). Smokers and those consumed alcohol each constituted 3%. There were 4% who were diabetics.

**Table 2:** Characteristics of household contacts of pulmonary TB patients screened for TB (N = 2150).

Characteristics	n %
Gender	
Male	866 (40)
Female	1284 (60)
Average age [Median (IQR)]	30 (18, 45)
Age group (years)	
< 6	109 (5)
> = 6	2041 (95)
Body mass index (BMI) [Median (IQR)] *	22.1 (18.9, 25.7)
On current TB treatment	4 (<1)
Known diabetic	81 (4)
Known HIV reactive	1 (<1)
Smoking present	54 (3)
Alcohol intake present	58 (3)
On immunosuppressive drugs	15 (1)
Taken TB treatment in the past	23 (1)
Currently pregnant	6 (<1)

* BMI available only for 713 contacts.

### TB screening in household contacts

The main modality of screening for TB in HHC was symptom screening and sputum smear examination which was done for 1095 (51%) (Table 3). Symptom screening and sputum CBNAAT test was done for 204 (10%). Chest radiograph was done for 372 (17%). There were 21 HHC diagnosed with PTB. Of the 21, there were 9 males, median age was 25 years, 3 were diabetics and 2 had TB treatment in the past. Of the 21 contacts, TB was microbiologically confirmed in 14 contacts. There were 13 HHC who were CBNAAT positive with rifampicin resistance not detected.

**Table 3:** Details of TB screening among household contacts of pulmonary TB patients (N = 2150).

TB screening details		N = 2150	
		n	%
**Screening method**	Only symptom screening	298	14
	Symptom screening + Chest x-ray	162	8
	Symptom screening + smear examination	1095	51
	Symptom screening + CBNAAT (Xpert MTB/RIF)	204	10
	Symptom screening + Chest x-ray + smear examination	88	4
	Symptom screening + Chest x-ray + CBNAAT (Xpert MTB/RIF)	24	1
	Symptom + Smear + CBNAAT (Xpert MTB/RIF)	181	8
	Symptom screening + Chest x-ray + smear examination + CBNAAT (Xpert MTB/RIF)	98	4
**Screening method result**			
Symptom screening	Presumptive TB symptoms	71	3
Chest x-ray (n = 372)	Normal	366	98
	Abnormal	6	2
Smear examination (n = 1462)	Positive	11	1
	Negative	1451	99
CBNAAT (Xpert MTB/RIF) (n = 507)	Positive	13	3
	Negative	494	97
Tuberculin skin test (TST) (n = 123)	Positive	9	7
	Negative	114	93

### Reasons for household contacts not screened for TB at baseline

Non-listing of HHC was observed in 96 (7%) of 1364 index PTB patients initiated on treatment during the implementation phase. The main reasons were HCW related for 68 (70%) due to temporary/permanent non-availability of HCWs mainly TB-HV or increased work load (Table 4). Index patient related reasons in 28 (30%) PTB patients were non-co-operation, death and lost to treatment.

**Table 4:** Reasons for household contacts of pulmonary TB patients not screened for TB.

Reasons for household contacts not screened for TB		n	%
Non-listing of household contacts of PTB patients (N = 96)	Index patient related		
	Not co-operative	12	13
	Died	5	5
	Lost to treatment	5	5
	Shifted to private provider	4	4
	Transferred for treatment	2	2
	Health care worker related		
	Non-availability	35	36
	Others (work load, no documentation)	33	34
Non-screening of listed household Contacts (N = 537)	Household contact related		
	Feeling healthy	199	37
	Inconvenient time	109	20
	Out-station visit / resident	52	10
	Stigma	37	7
	Poor awareness	20	4
	Health issues	14	3
	Old age	13	2
	No response	55	10
	Index patient related		
	Unwilling	24	4
	Lost to treatment	7	1
	Death	7	1

The main reasons for listed HHC not being screened for TB include feeling healthy (37%), time inconvenience (21%), outstation visit / resident (10%) and stigma (7%) (Table 4).

### Follow-up TB screening in household contacts

Information on 6^th^ and 12^th^ month follow-up of HHC is provided in Table 5. Of the eligible 2128 HHC, 1403 (70%) and 1064 (50%) underwent 6^th^ month and 12^th^ month follow-up TB screening. There were 1019 (48%) contacts who were screened for TB at both 6^th^ and 12^th^ month follow-up while 384 (18%) and 45 (2%) were screened only at 6^th^ and 12^th^ month respectively. Two TB cases were diagnosed during the follow-up period.

**Table 5:** Follow-up TB screening in household contacts of pulmonary TB patients.

	6^th^ month	12^th^ month
	n (%)	n (%)
Number of household contacts eligible for follow-up	2128	2127
Household contacts screened for TB	1403 (70)	1064 (50)
Household contacts with TB symptoms	7	34
Household contacts diagnosed with TB	1	1
Household contacts started on anti-TB treatment	1	1
Number of household contacts not screened for TB	725 (30)	1063 (50)
Reasons for household contacts not screened for TB		
Household contact related	166 (23)	330 (31)
Feeling healthy	25	37
Inconvenient time	25	0
Stigma	1	0
Migration	12	38
Out-station visit	52	50
Not interested/unwilling	40	184
Untraceable	3	21
Died	3	-
Lack of awareness	5	-
Index case related	66 (9)	6 (1)
Died	10	2
Lost to treatment	56	4
Health care worker related	493 (68)	727 (68)
Non-availability	88	82
Lack of time	364	627
No information	41	18

Reasons for non-adherence to follow-up TB screening were primarily due to HCW non-availability or lack of time to follow-up in nearly 60% of the HHC (Table 5).

### Excerpts from Focus group discussion among the health care workers

The participants for the FGD included 24 TB-HVs and 6 STS who were representative of a total of 56 TB-HVs and 24STS in the 15 Zones of Chennai District. The participants who were trained on the usage of HHC card and register were randomly selected from the line-list and written informed consent was obtained prior to FGD. The mean years of service of the FGD participants in the TB program was 7.5 years (range 2 to 17 years).

#### Usefulness of the Household Contact card and register

Most of the HCW felt that the card was useful to keep track of contacts with individual attention

‘Earlier we used to miss out on contacts as we were not able to keep track of all the contacts for all the index cases. Now with this card in place we are able to document all the contacts of the index cases’

‘This card has helped us to know all the contacts of all the patients by name when we ask about them to the index they feel happy that we are concerned on them and that helps to build a rapport with them’

With regard to the difficulties in collecting data the responses highlighted were

‘The card is very elaborate with too many entries to make. The documentation work is very difficult for us’

‘Collecting details for BMI which includes weight and height of all contacts is very difficult’

‘When there are more number of contacts in a family we have to maintain multiple cards for the same family. As there are more entries to make filling up these cards is very difficult’

Few expressed preference to register instead of card was mentioned by some

‘Instead of this card, register is comfortable because register we can document all the cases together and keeping track will be easy if we just run through the register to know the contacts that are due for screening’

#### Reasons for not screening household contacts for TB at baseline

Majority of the HCW’s felt that Stigma, lack of time, financial constraints and poor awareness as reasons for household contacts not being screened

‘The index patients who have not disclosed their status to their family members do not bring them for screening’

‘Loss of wages. Patients are not willing to lose their one day wages for this purpose. Transportation cost incurred’

‘School and college going contacts do not come for screening’

‘Women too do not agree easily for screening stating the reason that they are not at risk for TB as they do not have habits like smoking and alcohol’

Challenges with taking x-ray and screening of children were emphasized

‘Delay in taking the x ray and the time taken for reading the x rays. Getting x ray opinion from MO is very difficult’

‘Screening child contacts is even more challenging. Parents with child contacts take them to private practitioners to get their opinion and decide not to take up the screening or at time decline treatment also’

‘Especially with child contacts less than 6 years when we send them for IPT initiation they are asked to get admitted in hospital and sputum is collected through tube from their nose. The parents are very much scared about this procedure and do not agree for admission or treatment initiation’

#### Reasons for poor follow-up TB screening in household contacts

The long-term follow-up for HHC was considered challenging especially when PTB patients themselves do not co-operate for follow-up

‘Following up the contacts for 12 months is very difficult because the index on treatment itself may not cooperate for the follow up screening so with their contacts it is even more difficult’

#### Recommendations to improve household contact card and register

Minimal documentation was requested in HHC card and registers

‘The number of entries has to be reduced with minimal information to fill like their basic demographic with screening done date and due date’

#### Recommendations to improve household contact screening for TB

The importance of counselling was highlighted to improve contact screening

‘Family counseling is the best solution to improve contact tracing counseling all the family members will improve the cooperation from all the contacts’

‘The more we counsel and motivate the contacts they turn up for the screening. It largely depends on the way the staff motivate the patients and the contacts for screening’

Additional staff for screening activity was suggested

‘More human resource is needed to do this contact screening task’

‘It would be great if a separate staff is appointed to look after this contact screening and tracing them for follow up as this will reduce the work load of the HV’s’

Interventions such as incentives, home visits, TST availability in health care centres were suggested to improve TB contact screening in household contacts

‘It would be good if the contacts can be provided with some incentives or any nutritional supplements which will motivate and encourage them to get tested’

‘Doing contact screening by going to their house is better because only when we go to their house we will come to know the actual number of contacts in that house and it will be easy for us to collect all the details required to fill all the entries’

‘TST can be tested in center itself for the children. We are sending them to Government hospital. So it takes the whole day. Because of this, they do not come the next time’

## Discussion

Systematic documentation by line-listing HHC of PTB patients and detailed TB screening using HHC card and register significantly improved contact screening from 36% to 80%. A study in Ethiopia which used the Family & Household TB Contact Screening Logbook documented contact screening in 55.7% of TB patients [9]. Earlier studies in Ethiopia under field and institutional setting reported 33.7% and 45.7% adherence to contact screening respectively [5, 10]. The HCWs in our study felt that the HHC card/register was a useful tool for contact screening. However, they preferred the entries in the HHC card/register to be limited to demographic details and TB screening status. The global target of TB preventive treatment to at least 30 million people in 2018–2022 includes 24 million HHC [11]. HHC screening for TB is the initial step to initiate TB preventive therapy. The study has documented that activity of contact screening could be improved under TB program settings by systematic line-listing of HHC using HHC card/register.

There were 1% of HHC diagnosed with TB. An earlier study from Maharashtra documented TB prevalence of 1.15% among 521 HHC of newly diagnosed sputum smear (SS) positive TB index cases [12]. However, a clinic based study in Chennai documented TB in 5.3% (29 out of 544) of HHC [13]. A retrospective study in 1052 HHC of newly diagnosed TB patients in urban TB clinic in Delhi documented active TB disease in 4.3% [14]. TB case detection depends largely on the TB screening strategy. In our study, the most common strategy used was symptom screening and sputum smear examination in 51% of contacts. The use of chest radiograph in combination with symptom screening has been recommended in a previous study for new TB case yield [13]. However, the availability of chest radiograph including its interpretation poses huge challenge and delay in contact screening according to the HCWs as highlighted during the FGDs. Since sputum smear examination is readily available at the health facility, this test seems to be preferred for TB screening based on feasibility. Previous study from Delhi among HHC of TB patients reported that significant number of co- prevalent (84.6%) and incident TB (70.9%) to be smear negative but culture positive leading to under-diagnosis of TB [15]. CBNAAT was performed in 507(19%) of 2687 HHC in the present study. The integrated approach of TB contact tracing by special arrangement for chest radiograph, sputum and gene Xpert MTB/RIF examination yielded a high TB detection rate of 13.8% in Myanmar [16]. In the present study, we attempted to observe and document the practice of HHC screening under program settings in the study area. The lack of uniformity of investigations among HHC and the low TB case detection needs to be explored in further studies.

IPT initiation in children aged less than 6 years improved from 34% to 59% with the use of HHC card/register. The challenges of contact screening for TB in children include referral and admission for gastric aspirate, TST availability, anxiety in parents and opinion from private providers. This underscores the importance of the building capacity of Medical Officer at the level of Primary health centres to evaluate children for TB, counseling of parents and involvement of private providers for IPT initiation.

HCW availability and co-operation is essential for HHC screening for TB. Specific staff for counseling and contact screening activity was suggested by the HCWs. HHC who feel they are healthy, lack of time, stigma, unwillingness of index TB patient are some reasons for non-adherence to contact screening observed in our study. Previous study from Ethiopia has documented that education level of Index patient, knowledge on TB, satisfaction with health care services, health education by HCWs influence contact screening practice [10]. A study from Thailand which documented 52% contact screening adherence among TB patients identified higher perceived susceptibility, lower perceived barriers, to influence contact screening [4]. Knowledge, attitudes and practices of contacts and TB patients influence contact investigation has been reported from a study in Vietnam [17]. The importance of counseling and incentives to HHC was highlighted by the HCWs in the FGD sessions to improve contact screening.

Follow-up of HHC for a period of one year was observed to be challenging both from the provider and the HHC perspective under Program settings since information on follow-up could be obtained in half of the HHC. We observed 2 contacts with incident TB during one year follow-up period which was based primarily on TB symptom screening. An earlier study from Delhi reported incident TB cases of 2.6% over a 24 month follow-up period [15].

We did not obtain the perceptions of patients or their contacts on screening for TB in the present study. The details of co-morbidity in HHC were self-reported. The views on the usefulness of the HHC card and register by FGD was from about one-third of the HCWs who used them. The inherent limitations of FGDs need to be considered though all the required procedures were followed in conducting them.

This study has demonstrated that Household contact card and register with detailed line-listing of HHC is a useful tool for the HCWs to ensure TB screening in HHC of PTB patients. The challenges identified in contact screening under TB program settings needs to be addressed to achieve optimal TB contact screening.

## Supporting information

S1 File Household contact card. (PDF)

S2 File Household contact register. (PDF)
