# Supplementary material for: Tuberculosis screening in household contacts of pulmonary tuberculosis patients in an urban setting
Source: PLoS One. 2020 Oct 15;15(10):e0240594. doi: 10.1371/journal.pone.0240594 (PMC7561118; doi:10.1371/journal.pone.0240594)
Supplement: S1 File — (PDF) [file pone.0240594.s001.pdf]

| Name of TU: | TU No: |
|---|---|

# HOUSEHOLD CONTACT CARD

## Index TB patient details

| Name of patient | TB No | MDR-TB treatment | Date of start of treatment | Sputum status | Number of household contacts |
|---|---|---|---|---|---|
| | | Yes / No | | Pos/Neg | |

## Household contact – Baseline TB screening

| | | | | | | | |
|---|---|---|---|---|---|---|---|
| Contact screening no | | | | | | | |
| Name of contact | | | | | | | |
| Age in years / months if < 1 year | | | | | | | |
| Gender | | Male / Female | | Male / Female | | Male / Female | |
| Mobile/Landline number | | | | | | | |
| Current TB treatment | | Yes / No | | Yes / No | | Yes / No | |
| Known Diabetic | Duration (yrs) | Yes / No | | Yes / No | | Yes / No | |
| Known HIV reactive | Duration (yrs) | Yes / No | | Yes / No | | Yes / No | |
| Smoking | Duration (yrs) | Yes / No | | Yes / No | | Yes / No | |
| Alcohol intake | Duration (yrs) | Yes / No | | Yes / No | | Yes / No | |
| On immunosuppressive drugs | | Yes / No | | Yes / No | | Yes / No | |
| TB treatment in the past | | Yes / No | | Yes / No | | Yes / No | |
| Currently pregnant | | Yes / No/ NA | | Yes / No/ NA | | Yes / No/ NA | |
| | | | | | | | |
| Contact screened for TB | | Yes / No / NA | | Yes / No / NA | | Yes / No /NA | |
| Reason if 'no' | | | | | | | |
| Date of screening | | | | | | | |
| Presumptive TB symptoms | | Yes / No | | Yes / No | | Yes / No | |
| Body weight in Kgs /Height in cm | | | | | | | |
| BMI | | | | | | | |
| | | | | | | | |
| *To be filled-in by Medical Officer* | | | | | | | |
| Chest x-ray | | Normal/Abnormal/ND | | Normal/Abnormal/ND | | Normal/Abnormal/ND | |
| Sputum /GA - Smear | | Positive/Negative/ND | | Positive/Negative/ND | | Positive/Negative/ND | |
| Sputum /GA - Xpert MTB/RIF | | Positive/Negative/ND | | Positive/Negative/ND | | Positive/Negative/ND | |
| Others –Investigations (Culture/FNAC/USG/HPE etc) | | | | | | | |
| Tuberculin Skin Test (TST) | | Positive/Negative/ND | | Positive/Negative/ND | | Positive/Negative/ND | |
| Screening outcome | | TB / Not suggestive of TB / NK | | TB / Not suggestive of TB / NK | | TB / Not suggestive of TB / NK | |
| Reason if 'Not Known' | | | | | | | |
| Started on ATT | | Yes / Eligible not started /Not required | | Yes / Eligible not started /Not required | | Yes / Eligible not started /Not required | |
| Started on IPT | | Yes / Eligible not started /Not required | | Yes / Eligible not started /Not required | | Yes / Eligible not started /Not required | |
| Remarks | | | | | | | |
| Signature of Medical Officer & date | | | | | | | |

ND: Not done; NA: Not applicable; NK: Not known; GA: Gastric Aspirate

**Household contact – Follow-up TB screening [Scheduled visit at 6 months and 12 months]**

| | | | |
|---|---|---|---|
| **Contact screening no** | | | |
| **Follow-up screening due date** | | | |
| **Contact screened for TB** | **Yes / No** | **Yes / No** | **Yes / No** |
| **Reason if 'no'** | | | |
| **Follow- up screening done date** | | | |
| **Presumptive TB symptoms** | **Yes / No** | **Yes / No** | **Yes / No** |
| ***To be filled by Medical Officer*** | | | |
| **Chest x-ray** | **Normal/Abnormal/ND** | **Normal/Abnormal/ND** | **Normal/Abnormal/ND** |
| **Sputum /GA - Smear** | **Positive/Negative/ND** | **Positive/Negative/ND** | **Positive/Negative/ND** |
| **Sputum /GA - Xpert MTB/RIF** | **Positive/Negative/ND** | **Positive/Negative/ND** | **Positive/Negative/ND** |
| **Others –Investigations (Culture/FNAC/USG/HPE etc)** | | | |
| **Screening outcome** | **TB / Not suggestive of TB / NK** | **TB / Not suggestive of TB / NK** | **TB / Not suggestive of TB / NK** |
| **Reason if 'Not Known'** | | | |
| **Started on ATT** | **Yes / Eligible not started /Not required** | **Yes / Eligible not started /Not required** | **Yes / Eligible not started /Not required** |
| **Signature of Medical Officer & date** | | | |
| | | | |
| **Follow-up screening due date** | | | |
| **Contact screened for TB** | **Yes / No** | **Yes / No** | **Yes / No** |
| **Reason if 'no'** | | | |
| **Follow- up screening done date** | | | |
| **Presumptive TB symptoms** | **Yes / No** | **Yes / No** | **Yes / No** |
| ***To be filled by Medical Officer*** | | | |
| **Chest x-ray** | **Normal/Abnormal/ND** | **Normal/Abnormal/ND** | **Normal/Abnormal/ND** |
| **Sputum /GA - Smear** | **Positive/Negative/ND** | **Positive/Negative/ND** | **Positive/Negative/ND** |
| **Sputum /GA - Xpert MTB/RIF** | **Positive/Negative/ND** | **Positive/Negative/ND** | **Positive/Negative/ND** |
| **Others –Investigations (Culture/FNAC/USG/HPE etc)** | | | |
| **Screening outcome** | **TB / Not suggestive of TB / NK** | **TB / Not suggestive of TB / NK** | **TB / Not suggestive of TB / NK** |
| **Reason if 'Not Known'** | | | |
| **Started on ATT** | **Yes / Eligible not started /Not required** | **Yes / Eligible not started /Not required** | **Yes / Eligible not started /Not required** |
| | | | |
| **Final outcome at One year** | **No TB / TB / Lost** | **No TB / TB / Lost** | **No TB / TB / Lost** |
| **Reason for 'Lost'** | | | |
| **Remarks** | | | |
| **Signature of Medical Officer & date** | | | |

**ND: Not done; NK: Not known; GA: Gastric Aspirate**
