# Supplementary material for: Tuberculosis screening in household contacts of pulmonary tuberculosis patients in an urban setting
Source: PLoS One. 2020 Oct 15;15(10):e0240594. doi: 10.1371/journal.pone.0240594 (PMC7561118; doi:10.1371/journal.pone.0240594)
Supplement: S2 File — (PDF) [file pone.0240594.s002.pdf]

HOUSEHOLD CONTACT REGISTER [Left side – page]

S.No	Name of Index patient	TB.No	Date of start of treatment	Number of household contact	Number of contacts currently on ATT	Number of contacts screened	Contact screening number	Screening date	Name	Age	Gender [M/F]	BMI [Kg/m^2]

M: Male, F: Female

HOUSEHOLD CONTACT REGISTER [Right side – page]

DM [Y/N]	HIV R [Y/N]	Smoking [Y/N]	Alcohol [Y/N]	Immuno-suppressive Drugs [Y/N]	Past ATT [Y/N]	Currently Pregnant [Y/N/NA]	Baseline screening outcome [TB/No TB/NK]	6th month screening date	6th month screening outcome [TB/No TB/NK]	12th month screening date	12th month screening outcome [TB/No TB/NK]	Final screening outcome [No TB/TB/ Lost]	Re-marks

Y: Yes, N:No, NA: Not applicable, NK: Not known
